# Impact of Chronic Obstructive Pulmonary Disease on Infectious Complications and Mortality in Patients With Aneurysmal Subarachnoid Hemorrhage

**DOI:** 10.3389/fneur.2021.723115

**Published:** 2021-11-12

**Authors:** Lan Yang, Yu Zhang, Wei Yao, Fang Fang, Weimin Li

**Affiliations:** ^1^Department of Respiratory and Critical Care Medicine, West China Medical School/West China Hospital, Sichuan University, Chengdu, China; ^2^Department of Neurosurgery, West China Medical School/West China Hospital, Sichuan University, Chengdu, China; ^3^Department of Neurosurgery, Affiliated Hospital of Chengdu University, Chengdu, China; ^4^Department of Orthopedics, Dandong Central Hospital, China Medical University, Shenyang, China

**Keywords:** intracranial aneurysm, chronic obstructive pulmonary disease, subarachnoid hemorrhage, risk factor, prognosis

## Abstract

**Background and Purpose:** Chronic obstructive pulmonary disease (COPD) has been associated with several complications and mortality in acutely ill patients. For patients with aneurysmal subarachnoid hemorrhage (aSAH), the association between COPD and clinical outcomes remains unclear.

**Methods:** In this retrospective cohort study, we analyzed consecutive aSAH patients admitted to the West China Hospital between 2014 and 2019. Propensity score matching analysis and cox regression models was used to assess the association between COPD and mortality. The primary outcome was long-term mortality.

**Results:** Using a clinical database from a large university medical center, 2,925 patients with aSAH were identified, of whom 219 (7.5%) also had COPD. Patients were followed-up for a median of 3.8 years, and during follow-up 633 patients (21.6%) died. Long-term mortality was higher in patients with COPD compared to patients without COPD in the Cox regression models [adjusted hazard ratio (HR) 1.52, 95% confidence interval (CI) 1.14–2.02]. Propensity score matching analysis also showed similar associations between COPD and mortality in hospital, at 1 year, at 2 years, and at long-term. Similarly, patients with COPD had significantly higher incidence of infections, such as pneumonia [odds ratio (OR) 3.24, 95% CI 2.30–4.56], urinary tract infection (OR 1.81, 95% CI 1.20–2.73), bloodstream infection (OR 3.83, 95% CI 1.84–7.99), and hospital infection (OR 3.24, 95% CI 2.28–4.61).

**Conclusions:** Among aSAH patients, COPD is associated with increased mortality. COPD represents a significant risk factor for infections. Given that these are preventable complications, our findings are of clinical relevance.

## Introduction

Chronic obstructive pulmonary disease (COPD) is the third leading cause of death in the world (1–3) and is currently characterized by systemic involvement and multiple comorbidities (4). Growing evidence indicated that COPD independently predicts mortality and morbidity in patients undergoing surgery and patients with critically ill (5–8). However, the impact of COPD on outcomes in patients with aneurysmal subarachnoid hemorrhage (aSAH) remains unclear (9). Only one observational study has addressed the association between COPD and mortality in patients with aSAH (10). That study demonstrated that COPD did not increase in-hospital mortality after adjusting confounders. The published literature is sparse with respect to the long-term mortality of patients with COPD after aSAH.

Moreover, there is no data identifying the impact of COPD on infectious complications in patients with aSAH. The question of the potential impact of COPD on infectious complications in patients with aSAH is important because if COPD was indeed associated with infections, patients with COPD after aSAH would benefit from prophylactic antibiotics. A Cochrane review concluded that use of prophylactic antibiotics results in a benefit in reducing exacerbations in COPD patients (11). However, evidence from two large randomized clinical trials did not found the benefits of use of prophylactic antibiotics in lower risk of pneumonia or death for patients with stroke (12, 13). A possible explanation for the failure is that the included patients in the trials have low risk of infection, with 7 and 16% patients developing pneumonia, respectively. For patients with aSAH, about 20% of them develop pneumonia (14). COPD is also one of the most frequent comorbid conditions and a risk factor for developing pneumonia in critically ill patient (15).

With the increasing global incidence of COPD (16) and its high prevalence in patient with aSAH (10), we assessed the impact of COPD on outcomes in patient with aSAH, using propensity score matching (PSM) to form groups for comparison with near-identical distributions of background and potential confounder variables.

## Materials and Methods

### Study Design

We performed a retrospective cohort study. We consecutively evaluated the electronic health record of patients with aSAH admitted to the West China Hospital, Sichuan University, from January 2014 to June 2019, which is the largest hospital in Sichuan province, with a population of 91 million. This study was approved by the ethics committee of West China hospital (No. 20191133). The ethics committee has exempted written informed consent of patients included in the study because this study posed minimal-risk research and used only observational data.

### Study Population

Patients were eligible if they had an intracranial aneurysm identified by imaging in the presence of SAH. Intracranial aneurysms were identified by cerebral angiography, MRA, CTA, or operation. SAH was confirmed with neuroimaging (including CT, MRI, or angiography), cerebrospinal fluid analysis, or intraoperatively by a neurosurgeon.

Participants were excluded in case of aneurysms related to trauma, arteriovenous malformations, fusiform aneurysms, or non-definitive aneurysms, aneurysms that were treated before the presentation, or trauma SAH. Moreover, we also excluded patients whose household registration was not in Sichuan province or whose personal identification number was not found in the electronic medical record system, because we used personal identification number to identify mortality by searching the databases of the Household Registration Administration System in Sichuan province.

### Demographics Characteristics

The primary exposure was COPD. Diagnosis of COPD was based on medical reports. Demographic and clinical data included age, sex, hypertension, diabetes mellitus, coronary heart disease, smoking (current, ever, never), alcohol use, size of aneurysm, location of aneurysm, external ventricular drain, and treatment of aneurysm. Hunt & Hess grade and Fisher grade were also obtained on admission.

### Outcomes

The primary outcome was long-term mortality, which was defined as the mortality at the longest follow-up. The time of death was determined by searching the data bases of the Household Registration Administration System. In China, every resident has a unique identification number. If one dies, a death certificate should be reported to the household registration offices in the bureau of public security within 30 days as required by law. As the death certificate database is accurate and complete, the rate of loss to follow-up of our cohort was negligible.

Secondary outcomes included mortality in hospital, 1 year, and 2 years, neurological complications, infectious complications, acute kidney injury, length of hospital stay, and poor functional outcome at the time of discharge. Infectious outcomes were pneumonia, intracranial infection, urinary tract infection, and bloodstream infection. Neurological complications were hydrocephalus, delayed neurological ischemic deficits, rebleeding, and seizures.

Pneumonia is defined as a state of lung tissue inflammation of infectious etiology with the radiographic demonstration of parenchymal disease. Bloodstream infection was defined as positive blood culture necessitating treatment with antibiotics. Urinary tract infection was a positive urine culture or positive leukocyte esterase and positive nitrite on a urinalysis that necessitates treatment with antibiotics. Intracranial infection was defined as a positive cerebrospinal fluid culture requiring treatment with antibiotics. Poor functional outcome was defined as modified Rankin Scale (mRS) 4-6. Re-bleeding was defined as acute worsening in neurologic status along with an increase in hemorrhage volume which was confirmed in a repeat CT or MRI scan. Delayed ischemic neurological deficits was defined as angiographic vasospasm associated with a decline in neurological status lasting >2 h and with other causes being ruled out. Infections were diagnosed by treating physicians.

### Statistical Analysis

We used SPSS, version 24 (SPSS Inc) and R software version R3.3.2 (Matching and Frailty pack packages, R Foundation for Statistical Computing) for statistical analyses.

From our experience and from previous reports, age, sex, hypertension, diabetes mellitus, chronic renal failure, coronary heart disease, smoking, alcohol use, Hunt and Hess grade and Fisher grade were considered important confounders. Propensity score matching (17) was used to minimize bias from confounding variables when comparing patients with COPD and patients without COPD in the cohort study. The propensity score for each patient was calculated through the logistic regression modeling. Exact matching was performed in patients with and without COPD in a 1:5 ratio, with a caliper size of 0.2. We then compared the characteristics of patients with and without COPD using absolute standardized differences, and a difference more than 0.1 is considered meaningful.

We also adjusted potential confounding factors using logistic regression. Each variable was screened by univariable logistic regression model for each outcome. Variables with a *p*-value < 0.10 were entered into the multivariable logistic regression model which was created using backward elimination.

The Kaplan–Meier curve was generated for mortality. The relationships between COPD and long-term mortality were further assessed with pre-specified Cox regression models with adjustment for confounder using backward elimination.

For proportional outcomes comparing patients with COPD and patients without COPD after PSM, the paired *t*-test was used for continuous variables, and univariable logistic regression was used for binary variables. Two-sided *P* < 0.05 was considered statistically significant.

We used the E-value to assesses how large the effect from unmeasured confounding would be to negate the study results (18). E-values were computed with an online E-value calculator (https://mmathur.shinyapps.io/evalue/) (19).

## Results

**Figure 1:**
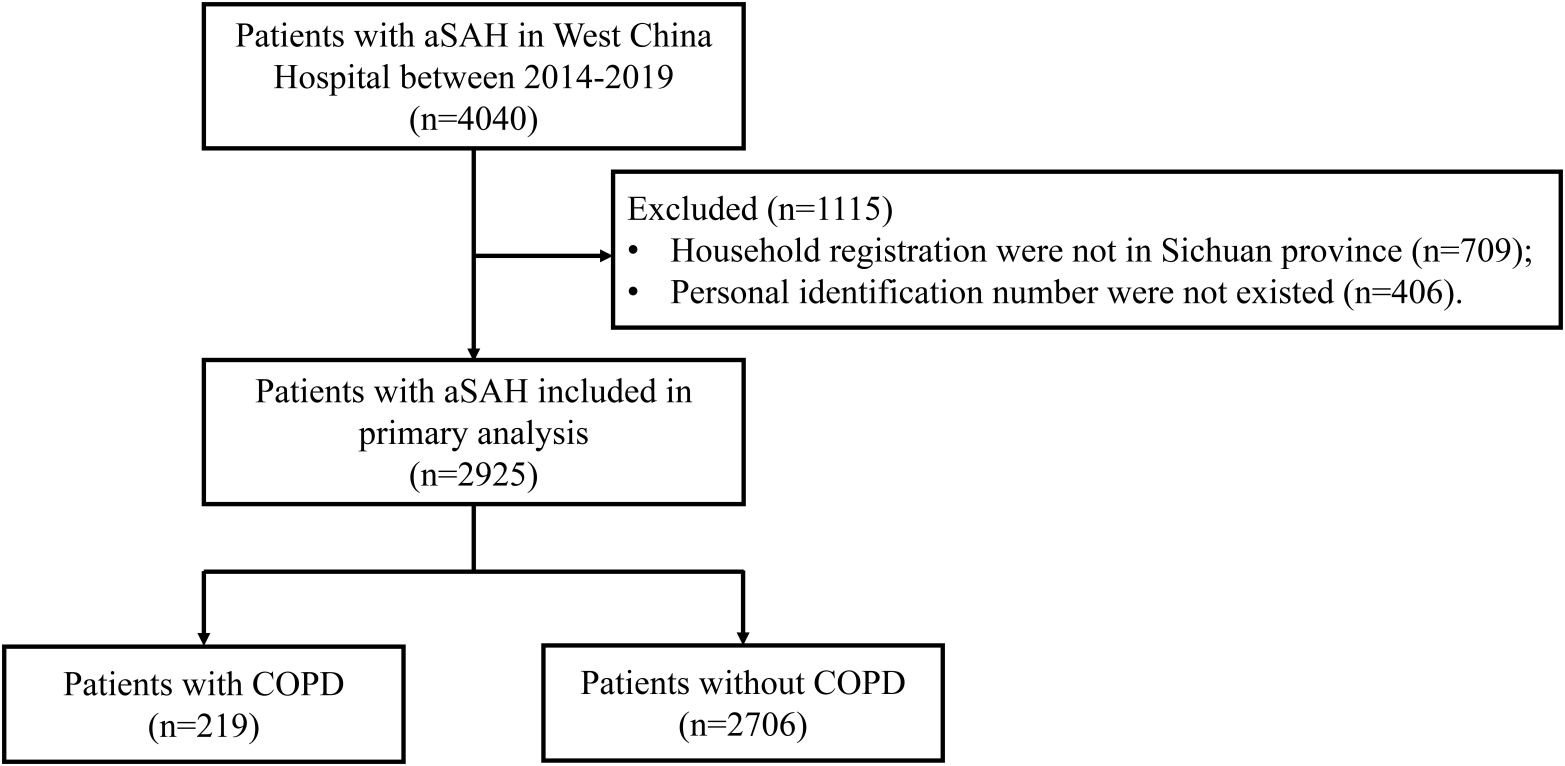
Flow chart of enrollment.

We screened 4,040 consecutive individuals with aSAH in West China hospital during the study period. After excluding 709 patients whose household registration were not in Sichuan province and 406 patients whose personal identification number were not existed in electronic medical record system, a total of 2,925 patients were included in this study (Figure 1). In patients with aSAH, 219 (7.5%) patients had COPD. Patient demographics stratified by COPD are shown in Table 1. Before matching, there were more old patients in the COPD group than in the non-COPD group. Compared with patients without COPD, patients with COPD more frequently had diabetes, and more patients with COPD are smokers. Patients with COPD have higher Hunt & Hess grade. There was a total of 166:830 matched pairs (1:5). After matching, the variables were balanced between patients with COPD and patients without COPD.

**Table 1:** Baseline characteristics of the patients by COPD.

**Characteristics**	**Before matching**			**After matching**		
	**Non-COPD (*n* = 2,706)**	**COPD (*n* = 219)**	**SMD**	**Non-COPD (*n* = 830)**	**COPD (*n* = 166)**	**SMD**
Mean age (SD), year	54.9 (11.7)	67.0 (9.1)	1.16	64.8 (8.9)	64.8 (8.8)	0.01
Female	1,771 (65.4)	125 (57.1)	0.17	491 (59.2)	97 (58.4)	0.02
Current Smoking	521 (19.3)	50 (22.8)	0.12	172 (20.7)	36 (21.7)	0.02
Alcohol abuse	516 (19.1)	48 (21.9)	0.07	174 (21.0)	31 (18.7)	0.06
Hypertension	683 (25.2)	60 (27.4)	0.05	243 (29.3)	44 (26.5)	0.06
Diabetes	147 (5.4)	20 (9.1)	0.14	68 (8.2)	13 (7.8)	0.01
Anterior circulation aneurysm	2,025 (74.8)	157 (71.7)	0.15	617 (74.3)	123 (74.1)	0.03
Mean size of aneurysm (SD), cm	0.7 (0.5)	0.7 (0.6)	0.03	0.7 (0.6)	0.7 (0.7)	0.04
**Hunt and Hess grade**						
I	262 (9.7)	16 (7.3)	0.11	69 (8.3)	11 (6.6)	0.01
II	1,392 (51.4)	109 (49.8)		404 (48.7)	85 (51.2)	
III	692 (25.6)	55 (25.1)		231 (27.8)	42 (25.3)	
IV	313 (11.6)	38 (17.4)		111 (13.4)	28 (16.9)	
V	47 (1.7)	1 (0.5)		15 (1.8)	0 (0)	
**Fisher grade**						
I	122 (4.5)	13 (5.9)	0.01	44 (5.3)	11 (6.6)	0.01
II	430 (15.9)	36 (16.4)		179 (21.6)	34 (20.5)	
III	345 (12.7)	22 (10.0)		121 (14.6)	20 (12.0)	
IV	1,253 (46.3)	106 (48.4)		486 (58.6)	101 (60.8)	
**Operation**						
Clip	1,710 (63.2)	123 (56.2)	0.21	491 (59.2)	98 (59.0)	0.04
Coil	337 (12.5)	21 (9.6)		85 (10.2)	15 (9.0)	
No treatment	659 (24.4)	75 (34.2)		254 (30.6)	53 (31.9)	
EVD	56 (2.1)	7 (3.2)	0.07	22 (2.7)	5 (3.0)	0.02

*COPD, chronic obstructive pulmonary disease; SMD, Standardized mean difference; SD, standard deviation; EVD, External ventricular drain*.

*Unless otherwise indicated, data are n (%)*.

The univariable logistic regression and multivariable logistic regression for the association between COPD and long-term mortality was shown in Supplementary Table 1. In univariate analysis, COPD was associated with increased odds of long-term mortality (OR 2.01, 95% CI 1.49–2.69). After adjusted for variables of age, hypertension, diabetes, size of aneurysm, external ventricular drain, and treatment of aneurysm in multivariable logistic regression, the association between COPD and long-term mortality was not changed (OR 2.01, 95% CI 1.49–2.69). Even after propensity score matching, our findings remained robust: COPD was associated with higher mortality (OR 1.63, 95% CI 1.02–2.62; Table 2). Propensity score matching analysis also showed similar associations between COPD and other mortality, such as in-hospital, 1 and 2 years.

**Table 2:** Comparison of unadjusted and risk-adjusted outcomes by COPD.

**Outcomes**	***n* (%)**	**Unadjusted**		**Multivariable regression adjustment**		**Propensity score adjustment**	
		**OR (95% CI)**	***P***	**OR (95% CI)**	***P***	**OR (95% CI)**	***P***
Mortality in hospital	148/2,936 (5.0)	2.15 (1.33–3.49)	0.002	1.98 (1.18–3.33)	0.01	1.86 (1.01–3.45)	0.05
Mortality at 1 year	358/2,936 (12.2)	1.74 (1.21–2.49)	0.003	1.41 (0.94–2.13)	0.10	1.59 (1.04–2.43)	0.03
Mortality at 2 years	485/2,823 (16.5)	2.17 (1.59–2.98)	<0.001	1.77 (1.22–2.58)	0.003	1.75 (1.20–2.55)	0.004
Long-term mortality	633/2,936 (21.6)	2.01 (1.49–2.69)	<0.001	1.46 (1.03–2.07)	0.03	1.45 (1.02–2.07)	0.04
mRS 4-6 at discharge	798/2,935 (27.2)	1.79 (1.35–2.38)	<0.001	1.85 (1.30–2.63)	0.001	1.59 (1.12–2.25)	0.01
mRS 4-5 at discharge	653/2,790 (22.2)	1.72 (1.26–2.34)	0.001	1.59 (1.08–2.36)	0.02	1.48 (1.02–2.16)	0.04
**Neurological complications**							
Hydrocephalus	293/2,936 (10.0)	1.36 (0.89–2.06)	0.15	1.03 (0.65–1.64)	0.89	0.99 (0.59–1.65)	0.97
Rebleeding	163/2,936 (5.6)	1.48 (0.88–2.49)	0.14	1.38 (0.81–2.34)	0.23	1.24 (0.63–2.45)	0.54
DNIDs	551/2,936 (18.8)	1.02 (0.72–1.45)	0.89	1.01 (0.71–1.44)	0.94	0.80 (0.51–1.26)	0.34
Seizures	103/2,936 (3.5)	1.83 (1.01–3.34)	0.05	2.26 (1.18–4.34)	0.01	2.06 (0.97–4.38)	0.06
**Infection complications**							
Pneumonia	807/2,936 (27.5)	4.32 (3.26–5.73)	<0.001	3.59 (2.63–4.89)	<0.001	3.24 (2.30–4.56)	<0.001
Intracranial infection	326/2,936 (11.1)	1.36 (0.91–2.02)	0.13	1.35 (0.89–2.05)	0.16	1.19 (0.73–1.93)	0.49
Urinary tract infection	429/2,936 (14.6)	1.94 (1.40–2.70)	<0.001	1.74 (1.22–2.47)	0.002	1.81 (1.20–2.73)	0.01
Bloodstream infection	91/2,936 (3.1)	2.99 (1.73–5.17)	<0.001	2.89 (1.67–5.02)	<0.001	3.83 (1.84–7.99)	<0.001
Hospital infection	1,110/2,936 (37.8)	3.90 (2.91–5.24)	<0.001	3.30 (2.41–4.51)	<0.001	3.24 (2.28–4.61)	<0.001

*OR, odds ratio; CI, confidence interval; DNIDs, Delayed Neurological Ischemic Deficits*.

**Figure 2:**
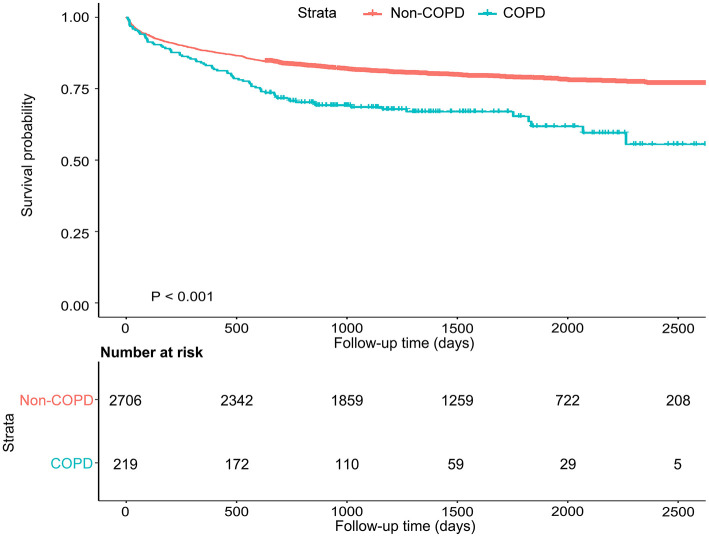
Kaplan–Meier curve of mortality after aSAH among patients with and without COPD.

Patients were followed-up for a median of 3.8 years, and during follow-up 633 patients (21.6%) died. The impact of COPD on mortality throughout follow-up period was shown in the Kaplan–Meier plot (Figure 2). Long-term mortality was higher in patients with COPD compared to patients without COPD in the Cox regression models [adjusted hazard ratio (HR) 1.52, 95% CI 1.14–2.02].

Before and after matching, COPD was associated with an increased risk of infectious complications, including pneumonia (OR 3.24, 95% CI 2.30–4.56), urinary tract infection (OR 1.81, 95% CI 1.20–2.73), bloodstream infection (OR 3.83, 95% CI 1.84–7.99), and hospital infection (OR 3.24, 95% CI 2.28–4.61). Before matching, COPD was associated with several neurological complications [hydrocephalus (OR 1.90, 95% CI 1.43–2.52), re-bleeding (OR 1.72, 95% CI 1.24–2.39), and seizures (OR 1.78, 95% CI 1.12–2.84)]. After matching, however, COPD was associated with an increased incidence of seizures, but not hydrocephalus and rebleeding. After matching, the length of hospital stay was significantly longer in patients with COPD (*P* < 0.001).

The E-value for long-term mortality (HR) was 2.01 with a lower limit of 1.42, suggesting that unmeasured confounding was unlikely to explain the findings.

**Figure 3:**
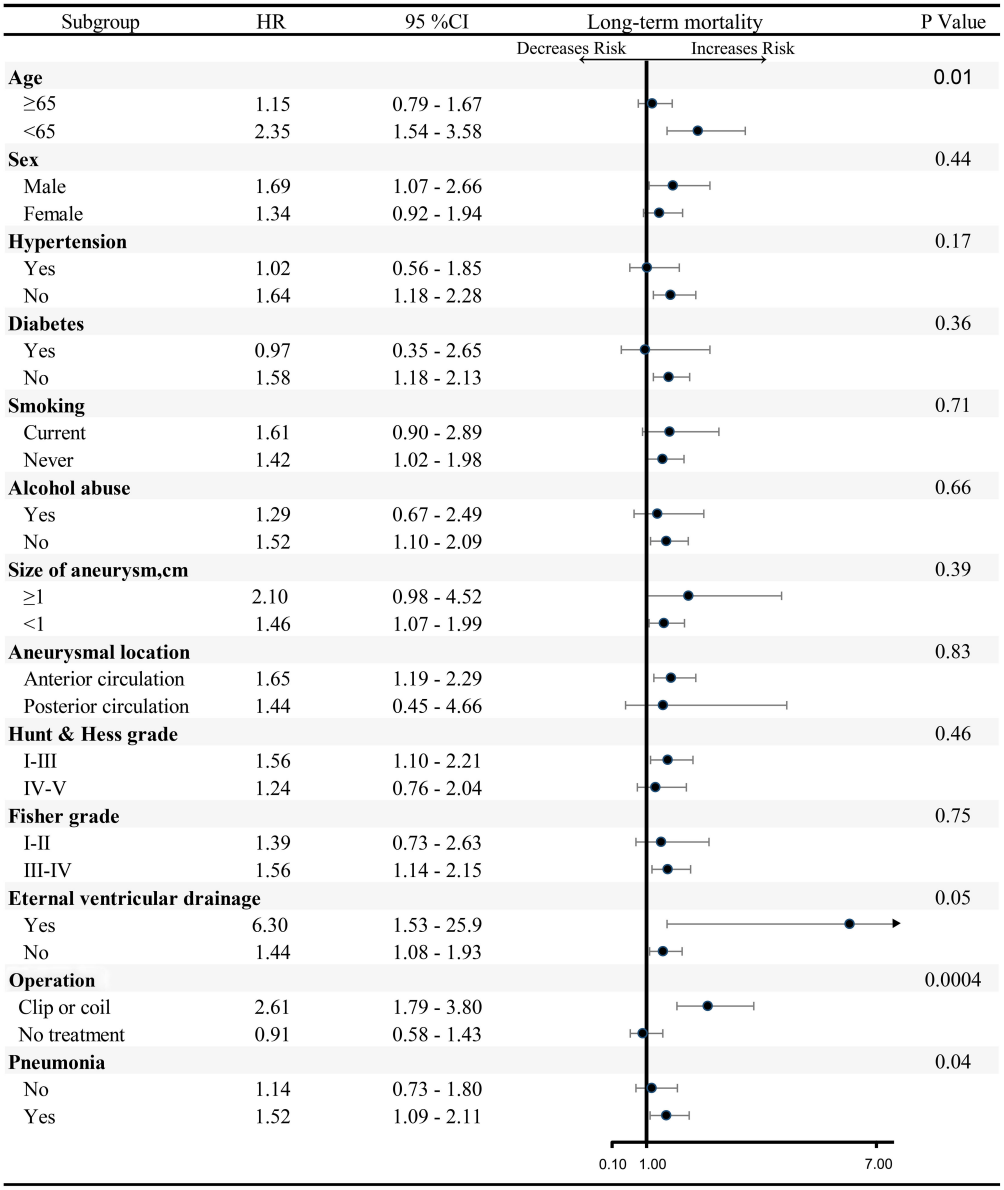
Subgroup analysis of association between COPD and long-term mortality. EVD, External ventricular drain; COPD, chronic obstructive pulmonary disease; HR, hazard ratio.

We further assessed interactions by other variables on COPD. Except for subgroup analyses of age, external ventricular drain, operation, and pneumonia, there is no significant effect modification of the change in COPD and long-term mortality on these variables (Figure 3).

## Discussion

In this cohort study of patients with aSAH, we found that compared to patients without COPD, patients with COPD have increased odds of short-term and long-term death and poor functional outcome at discharge. Moreover, COPD is associated with an increased incidence of seizures and infectious complications, especially pneumonia, which may contribute to the increased mortality observed in aSAH patients with COPD.

### Mechanisms

Several mechanisms may explain the association between COPD and poor outcomes. First, COPD causes spillover of multiple pro-inflammatory markers into the circulation, leading to chronic low-grade systemic inflammation, ultimately resulting in unstable plaque formation and prothrombotic events (20). Second, COPD, especially during exacerbation, are hypoxemic and hypercapnic at baseline which may increase their susceptibility to brain injury. The intraneural hypoxemia can occur in ~40–50% of patients with mild COPD (21). Third, COPD have associated comorbid conditions after stroke, such as seizure (22). Fourth, COPD are commonly treated with corticosteroids, and hospitalized patients on corticosteroids have a heightened risk of nosocomial infection.

### Mortality

For ischemic stroke patients, the association between COPD and the increased in-hospital mortality have been demonstrated both before (OR: 1.30, 95% CI: 1.26–1.35) and after adjusting confounders (OR: 1.08, 95% CI: 1.03–1.13) (10). Recently, the study by de Miguel-Díez et al. further confirmed this conclusion (23). However, for patients with aSAH, only one study related to this topic assessed the association between mortality and COPD in stroke patients (10). COPD was associated with increased odds of in-hospital mortality (OR 1.29, 95% CI 1.16–1.42) in univariate analysis; however, the association was not significant after adjusting confounders (adjusted OR 0.98, 95% CI 0.85–1.13) (10). The previous study was limited by short-term follow-up and the epidemiologic study design that was unadjusted for important confounders (e.g., hemorrhage severity), which led to the uncertainty of their conclusions.

### Functional Outcome

This study found an association of COPD with poor functional outcomes in patients with aSAH. While such an association has not been previously assessed in patients with aSAH, a study found that COPD increased the incidence of discharge to nursing homes and rehabilitation facilities after surgery (24), and another study found that the discharge destination is a surrogate for mRS functional outcome in stroke survivors (25). More research is needed to confirm the association of COPD with poor functional outcomes in patients with aSAH.

### Infection Complications

In this study, COPD was associated with an increased frequency of a variety of infection complications. In a cohort study by Lee et al., COPD is an independent risk factor for pneumonia and septic shock after total shoulder arthroplasty (26). Yakubek et al. published a study found that in patients undergoing total hip arthroplasty, patients with COPD are more likely to experience pneumonia and deep surgical site infection (24).

Two large randomized clinical trials conducted in patients hospitalized for stroke found that prophylactic antibiotics did not reduce the incidence of pneumonia (12, 13). A possible explanation for the lack of benefit is that the included patients have a general risk for pneumonia but not high risk, with 7–16% patients developing pneumonia in the control group. In the present study, half of the patients with COPD have pneumonia. The use of prophylactic antibiotics in patients with COPD may reduce the risk of progression to clinically overt pneumonia better than in general patients.

### Strengths and Limitations

One of the major strengths of our study is the high-quality, standardized, single-institution database, the large sample size, and the use of PSM to adjust for confounders. We determined all-cause mortality based on the household registration in systems, which is accurate and complete, without lost to follow-up.

However, the limitations of this study must also be considered. First, based on the retrospective study, the interpretation of the specific causal relationship for COPD on mortality was is limited. Secondly, pulmonary function testing was not recorded in our database. We cannot assess the association between severity of COPD and outcomes, limiting the strength of our conclusions. Moreover, the results of this study are contingent on the accuracy and reliability of COPD status data, which were based on patient self-report and family members of incapacitated patients. It is possible that some patients in the control group also had COPD, leading to reporting and recall biases; however, these biases may serve to increase the confidence in our conclusion.

## Conclusions

In aSAH patients, COPD was associated with a significant increase in short-term and long-term mortality. COPD increased the risk of infectious complications, especially pneumonia. Since these complications can potentially be prevented by antibiotics drugs, our findings are of clinical relevance and can open up new lines of inquiry. Certainly, future RCTs are needed to explore whether the use of prophylactic antibiotic therapy could improve the outcomes among these patients.

## Data Availability Statement

The original contributions presented in the study are included in the article/Supplementary Material, further inquiries can be directed to the corresponding author/s.

## Ethics Statement

The studies involving human participants were reviewed and approved by the Ethics Committee of West China Hospital. Written informed consent for participation was not required for this study in accordance with the national legislation and the institutional requirements.

## Author Contributions

FF and WL: study concept and design. YZ, LY, and WY: acquisition, analysis, or interpretation of data. YZ and WY: statistical analysis. YZ and LY: drafting of the manuscript. All authors: critical revision of the manuscript for important intellectual content.

## Funding

This research was funded by National Natural Science Foundation of China (grant number 81871890 and 91859203), National Key R&D Program of China (2018YFA0108604), the project of Health Commission of Sichuan province (19PJ003), the project of Sichuan Science and Technology Bureau (2020YFS0490), the 1.3.5 project for disciplines of Excellence-Clinical Research Incubation Project, West China Hospital, Sichuan University (21HXFH046), and Clinical Incubation Program of West China Hospital, SCU (2018HXFU008). The funders of the study had no role in study design, data analysis, data interpretation, or writing of the report.

## Conflict of Interest

The authors declare that the research was conducted in the absence of any commercial or financial relationships that could be construed as a potential conflict of interest.

## Publisher's Note

All claims expressed in this article are solely those of the authors and do not necessarily represent those of their affiliated organizations, or those of the publisher, the editors and the reviewers. Any product that may be evaluated in this article, or claim that may be made by its manufacturer, is not guaranteed or endorsed by the publisher.
