# Supplementary material for: Impact of Chronic Obstructive Pulmonary Disease on Infectious Complications and Mortality in Patients With Aneurysmal Subarachnoid Hemorrhage
Source: Front Neurol. 2021 Nov 12;12:723115. doi: 10.3389/fneur.2021.723115 (PMC8634885; doi:10.3389/fneur.2021.723115)
Supplement: Supplementary file 1 [file Table_1.DOCX]

Supplementary Material

# Supplementary Table

Table S1. Univariate and multivariate analysis for long-term mortality.

| Characteristics | Unadjusted | | Multivariable Regression Adjustment | |
| --- | --- | --- | --- | --- |
|  | OR (95% CI) | P | OR (95% CI) | P |
| Demographics | | | | |
| Age, year, mean (SD) | 1.03 (1.03-1.04) | <0.001 | 1.02 (1.02-1.03) | <0.001 |
| Female, n (%) | 0.90 (0.75-1.08) | 0.25 |  |  |
| Current Smoking, n (%) | 0.87 (0.74-1.03) | 0.11 |  |  |
| Alcohol abuse, n (%) | 1.04 (0.83-1.30) | 0.74 |  |  |
| Medical history, n (%) | | | | |
| Hypertension | 1.20 (0.98-1.46) | 0.08 | 1.01 (0.80-1.26) | 0.94 |
| Diabetes | 1.36 (0.96-1.94) | 0.09 | 0.98 (0.65-1.46) | 0.90 |
| Aneurysm characteristics, n (%) | | | | |
| Anterior location | 0.96 (0.80-1.14) | 0.61 |  |  |
| Size of aneurysm | 1.54 (1.33-1.78) | <0.001 | 1.46 (1.23-1.73) | <0.001 |
| Hemorrhagic characteristics, n (%) | | | | |
| Hunt & Hess grade | 2.76 (2.47-3.07) | <0.001 | 2.30 (2.04-2.59) | <0.001 |
| Fisher grade | 1.50 (1.34-1.68) | <0.001 | 1.27 (1.13-1.43) | <0.001 |
| EVD, n (%) | 4.73 (2.86-7.84) | <0.001 | 3.49 (1.95-6.26) | <0.001 |
| Aneurysm treatment, n (%) | 0.33 (0.28-0.38) | <0.001 | 0.41 (0.35-0.49) | <0.001 |
| COPD, n (%) | 2.01 (1.49-2.69) | <0.001 | 1.46 (1.03-2.07) | 0.03 |

Abbreviation. OR: odds ratio; CI: confidence interval; EVD: external ventricular drain; COPD: chronic obstructive pulmonary disease.
